# Efficient strategy for constructing duck enteritis virus-based live attenuated vaccine against homologous and heterologous H5N1 avian influenza virus and duck enteritis virus infection

**DOI:** 10.1186/s13567-015-0174-3

**Published:** 2015-04-16

**Authors:** Zhong Zou, Yong Hu, Zhigang Liu, Wei Zhong, Hangzhou Cao, Huanchun Chen, Meilin Jin

**Affiliations:** State Key Laboratory of Agricultural Microbiology, Huazhong Agricultural University, Wuhan, 430070 China; College of Veterinary Medicine, Huazhong Agricultural University, Wuhan, 430070 China; Hubei Collaborative Innovation Center for Industrial Fermentation, Hubei University of Technology, Wuhan, 430068 China; College of Life Sciences, AnQing Normal University, AnQing, 246011 China

## Abstract

Duck is susceptible to many pathogens, such as duck hepatitis virus, duck enteritis virus (DEV), duck tembusu virus, H5N1 highly pathogenic avian influenza virus (HPAIV) in particular. With the significant role of duck in the evolution of H5N1 HPAIV, control and eradication of H5N1 HPAIV in duck through vaccine immunization is considered an effective method in minimizing the threat of a pandemic outbreak. Consequently, a practical strategy to construct a vaccine against these pathogens should be determined. In this study, the DEV was examined as a candidate vaccine vector to deliver the hemagglutinin (HA) gene of H5N1, and its potential as a polyvalent vaccine was evaluated. A modified mini-F vector was inserted into the gB and UL26 gene junction of the attenuated DEV vaccine strain C-KCE genome to generate an infectious bacterial artificial chromosome (BAC) of C-KCE (vBAC-C-KCE). The HA gene of A/duck/Hubei/xn/2007 (H5N1) was inserted into the C-KCE genome via the mating-assisted genetically integrated cloning (MAGIC) to generate the recombinant vector pBAC-C-KCE-HA. A bivalent vaccine C-KCE-HA was developed by eliminating the BAC backbone. Ducks immunized with C-KCE-HA induced both the cross-reactive antibodies and T cell response against H5. Moreover, C-KCE-HA-immunized ducks provided rapid and long-lasting protection against homologous and heterologous HPAIV H5N1 and DEV clinical signs, death, and primary viral replication. In conclusion, our BAC-C-KCE is a promising platform for developing a polyvalent live attenuated vaccine.

**Electronic supplementary material:**

The online version of this article (doi:10.1186/s13567-015-0174-3) contains supplementary material, which is available to authorized users.

## Introduction

Ducks are considered one of the most important waterfowl for its various usages in different aspects. In China and southeast Asia, duck farming is not only a traditional agribusiness for nourishment, but also critical for habiliment. However, this traditional business is seriously threatened by numerous pathogens, such as avian influenza virus (AIV), duck hepatitis virus, duck enteritis virus (DEV), and duck tembusu virus [1,2].

Waterfowl is considered a larger and key natural reservoir of influenza A viruses. It is currently known that almost all the subtypes can be isolated from waterfowl with the exception of the H13 and H16 subtypes [3-5]. Notably, a novel reassorting avian-origin influenza A (H7N9) virus has been isolated from the ducks of live poultry markets [6]. As of October 25, 2013, the virus had caused 137 human cases and 45 human deaths during both epidemic waves in China [7]. The highly pathogenic avian influenza virus (HPAIV) H5N1 is a potential pandemic threat that has caused global concern in many Asian countries, and the duck is believed to be the primary source of infection [2]. Since 2003, a total of 694 human beings have been infected with HPAIV H5N1, with fatality rates approaching 60% [8]. Although many measures have been taken to control AIV infection and transmission, AIV is still a huge threat to public health and the duck industry.

Under these circumstances, vaccination, as an adjunct for improving bio-security and stamping-out policies, contributes to protecting ducks against AIV infection [9]. Currently, conventional inactivated vaccines are largely used for routine preventative vaccination and target vaccination programs [10]. However, inactivated vaccine production is costly and time-consuming, and the oil emulsion adjuvant can cause severe adverse reactions [11]. Furthermore, the risk of contamination by avian pathogens in the egg supply or microbial contaminants during processing has previously jeopardized vaccine supplies [12]. Additionally, inactivated vaccines usually need several weeks to provide solid immune protection [13], which is a major limitation in emergency vaccination to establish a buffer zone. Considering the drawbacks aforementioned, alternative vaccine manufacturing strategies are needed.

Duck viral enteritis is caused by the DEV which belongs to *Anatid herpesvirus* 1; it is an acute, contagious, and lethal disease of ducks, geese, and swans [14]. The DEV genome consists of approximately 160 kilobase pairs (kbp), each pair is composed of two unique sequences, unique long (UL) and unique short (US). The latter is flanked by inverted repeated sequences (IRS and TRS) [15]. A live C-KCE vaccine strain attenuated in the embryonated chicken egg has been developed and utilized to control duck viral enteritis for many years. Furthermore, the ability to induce DEV immunity is not significantly interfered by pre-existing antibodies [16]. Additionally, DEV possesses a wide tropism and can establish latency in the trigeminal ganglia, lymphoid tissues, and peripheral blood lymphocytes [17], in which they efficiently induce both strong humoral immune and cellular immune responses. Thus, the potential of C-KCE as a DNA-based platform for developing polyvalent vaccine deserves in-depth study.

Efficient genetic modification of herpesviruses, such as DEV, has come to rely on bacterial artificial chromosome (BAC) for generating recombinant viruses [18]. In this technology, a BAC-containing clone of the complete viral genome has to be generated, enabling propagation of the viral genome in *Escherichia coli* (*E.coli*) and avoiding the need for cumbersome cloning techniques [19]. Mating-assisted genetically integrated cloning (MAGIC) [20] utilizes bacterial mating, in vivo site-specific endonuclease cleavage and homologous recombination to catalyze the transfer of a DNA fragment between a donor vector in one bacterial strain and recipient plasmid in another separate bacterial strain. The recombination between these plasmids can be forced by inducing *I-Sce*I to site-specific cleavage and the *red*-*gam* recombinase to homologous recombination. Recombination events of MAGIC are genetically selected and result in placement of the gene of interest under the control of new regulatory elements with high efficiency [21].

In the present study, we established a BAC of the C-KCE strain. The hemagglutinin (HA) gene of HPAIV H5N1 was accurately inserted into the C-KCE genome based on MAGIC. A bivalent vaccine C-KCE-HA was generated by eliminating the BAC backbone via *Cre/Lox*p-mediated recombination [22]. Our data indicate that the HA gene inserted into that C-KCE genome was robustly expressed under the control of chicken β-actin promoter and cytomegalovirus immediate enhancer. We further demonstrated C-KCE-HA-immunized ducks induced both cross-reactive antibodies and T cell response against H5. Meanwhile this recombinant C-KCE-HA conferred 100% protection against two antigenically distinct strains of HPAIV H5N1 and virulent DEV challenge in the duck. Therefore, our BAC-C-KCE offers a suitable platform to generate polyvalent live attenuated vaccine against multiple pathogens.

## Materials and methods

### Virus strain and cells

The attenuated DEV C-KCE vaccine strain, obtained from the China Institute of Veterinary Drugs Control, was propagated and titrated in primary chicken embryo fibroblasts (CEF) propagated in Eagle’s minimal essential medium (EMEM, Biochrom), which was supplemented with 100 μg/mL penicillin, 100 μg/mL streptomycin, and 10% fetal bovine serum (FBS) at 37 °C under a 5% CO_2_ atmosphere. A virulent DEV strain (HB/10) isolated from Hubei Province in the central part of China was propagated and titrated in duck embryo fibroblasts (DEF). AIV H5N1 A/duck/Hubei/xn/2007 (H5N1) (XN/07) (clade 2.3.2) (GenBank accession number of HA: AHI43271.1) and A/duck/Hubei/HangMei01/2006 (HM/06) (clade 2.3.4) (GenBank accession number of HA: ACF16400.1) were propagated in the allantoic cavities of 10-day-old specific-pathogen-free (SPF) embryonated chicken eggs and stored at −80 °C.

### Plasmids and bacterial strains

All the plasmids and *E.coli* strains were kindly donated by Dr Lixin Ma. The mini-F plasmid pBlue-lox was maintained in *E.coli* strain DH10B-IS2 (umuC:araC-ParaBAD-I-Sce-I-FRT) which was constructed in Lixin Ma’s laboratory and expresses enzyme *I-Sce*I stimulated by 0.2% w/v L-arabinose [21]. The plasmid pML300 contained in DH10B-IS2 carries the *red*-*gam* recombinase gene stimulated by rhamnose, and is unable to replicate when the bacteria are grown at 42 °C [23]. DH10b was used for generating the recombinant donor plasmid pRThGA. DH10b, but not DH10B-IS2, provided a trans-acting factor π, which could support the conditional origin of replication from R6K, *ori*γ, which contained the donor vector plasmid pRThGA1-HA [24]. The plasmid pCAGGS-NLS/cre expressing Cre recombinase has been previously described [25].

### Generation of pBlue-lox-gB-UL26-Amp insertion plasmid and of donor plasmid pRThGA1-HA

**Figure 1:**
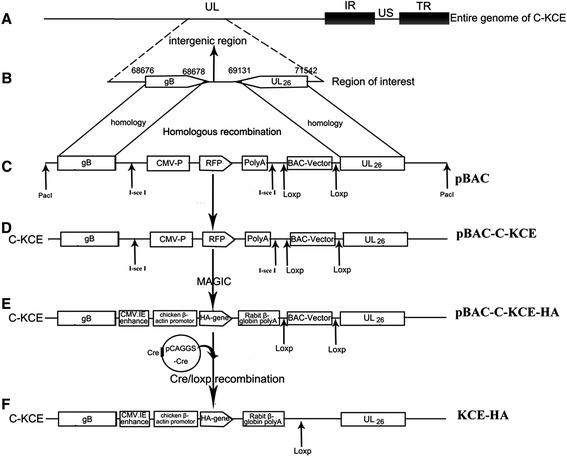
**Generation and characterization of H5 HA gene recombinant C-KCE-HA.** **(A)** The organization of the 158-kbp attenuated commercial DEV vaccine strain (C-KCE). **(B)** A portion of the genome C-KCE expanded to show the gB, UL26 gene and inter-genic region is depicted. **(C)** The organization of transfer vector pBlue-lox-gB-UL26-Amp digested by *Pac*I contains an enhanced red fluorescent protein gene and its expression cassette, two copies of the direct orientation 34-bp *Lox*p and two copies of the reverse complement 18-bp *I-sce*I. **(D)** After homologous recombination, pBlue-lox-gB-UL26 was inserted into the genome of C-KCE with the red fluorescent protein as a selection marker. **(E)** After MAGIC, the red fluorescent gene, CMV promoter, and ploy A were entirely replaced by the HA gene and its cassette. **(F)** After *Cre/Lox*p–mediate recombination, the BAC backbone was excised only 34-bp *Lox*p sequence positioned in C-KCE-HA genome.

The plasmid pBlue-lox-gB-UL26-Amp contains two copies of the *Pac*I restriction site, an enhanced red fluorescent protein (RFP) gene and its cassette, two copies of the direct orientation 34 bp *Lox*p, and two copies of the reverse complement 18 bp *I-sce*I. To insert the 8.28 kb spanning BAC mini-F plasmid into the C-KCE genome, a 454 bp inter-genic region [15] between the gB and UL26/UL26.5 genes was found to be suitable. The inter-genic region is flanked by two poly A sites (Figure 1B). The gB (UL27) is transcribed from left to right and its poly A site is located between nucleotides 65676 and 68678, whereas the UL26/UL26.5 genes are transcribed from right to left and their shared poly A site is located between nucleotides 69131 and 71254 on the complementary strand. Hence, for the insertion of the BAC plasmid sequence within the gB-UL26 junction region, a BAC insertion plasmid containing a *Pac*I insertion site within the gB-UL26 inter-genic region (nucleotides 68678 to 69131) and flanked by the upstream gB gene and the downstream UL26 gene was constructed. In brief, the gB upstream (partial) region and the inter-genic region were amplified as a 1.1 kb fragment using primers gB-F/gB-R (Table 1). The inter-genic region and downstream UL26 (partial) region were then amplified as a 1.2 kb fragment using primers UL26-F/UL26-R (Table 1). Briefly, the RFP gene under the control of the immediate early promoter of human cytomegalovirus (PHCMV) was amplified from pRTRA as a 1.8 kb fragment using primers Red-F/Red-R (Table 1). The three PCR products described above were used as the templates for “a ligation PCR” using primers UL26-F/Red-R (Table 1). A 4.3 kb fragment was then cloned into the *Sal* I/*Not* I sites of pBlue-lox, resulting in pBlue-lox-gB-UL26. To increase the copy number of pBlue-lox, the ampicillin resistance gene replicon fragment was amplified from pcdna3.1 (+) with the primers Amp-F/Amp-R (Table 1), and inserted into *Pac*I-digested pBlue-lox-gB-UL26 to obtain the plasmid pBlue-lox-gB-UL26-amp. Fragments from the CMV.IE enhancer to rabbit β-globin poly A were amplified from pCAGGS using the primers pCA-I-SceI-H1-F/pCA-I-SceI-H2-R flanked by 50-bp homology arms and *I-sce*I restriction sites. The fragment cut by *I-sce*I was ligated into the pRThGA vector (also cut by the same enzymes), resulting in the recombinant plasmid pRThGA1. The H5 gene was represented as AI A/duck/Hubei/2911/2007(H5N1) (GenBank ID: FJ784852.1) flanked by *Smal* I and *Xho* I restriction sites synthesized by Sangon Biotech Life Science Products & Services with several mutations at the cleavage site as previously described [26]. The fragment then was cloned into the *Smal* I and *Xho* I sites present in pRThGA1 to generate the donor vector plasmid pRThGA1-HA.

**Table 1:** **Primers used for generating pBAC-C-KCE, donor plasmid pRThGA and identification of the pBAC-C-KCE-HA**

**Purpose and primer**	**Sequence (5’ → 3’)**	**Sequence designation, restriction enzyme site and introduction sequence**
BAC insertion^a^		
UL26-F	aaa**gtcgac** *ataacttcgtatagcatacattatacgaagttat*gccgtatgaatgcgctgac	*Sal* I site (bold), *Lox* p sequence (italic)
UL26-R	**ttaattaa**cgcggacaaaacgacgattac	*Pac* I site (bold)
gB-F	gtaatcgtcgttttgtccgcg**ttaattaa**tgaaaaagacggcggtacaat	*Pac* I site (bold)
gB-R	aagaatgcattcggcctgg	
Red-F	ccaggccgaatgcattcttcgtggggtgtggtgcttttggt	
Red-R	tcga**gcggccgc** *tagggataacagggtaat*ccccaccttatatattctttcccaccct	*Not* I site (bold), *I-isce* I sequence (italic)
Amp-F	aaa**ttaattaa**ggggataacgcaggaaagaac	*Pac* I site (bold)
Amp-R	aaa**ttaattaa**acgtcaggtggcacttttcg	*Pac* I site (bold)
Modification pRThGA^b^		
pCA-*I-Sce*I-H1-F	aaa**tagggataacagggtaat** *gttgagcctttttgtggagtgggttaaattgtactagcgcgtttcgcttt*gcagtacatctacgtattagtcatcgctatta	*I-isce* I sequence (bold), Homology arm H1 (italic)
pCA-*I-Sce*I-H2-R	aaa**tagggataacagggtaat** *tagcatgcataacttcgtataatgtatgctatacgaagttatgcggccgc* cacacaggaaacagctatgaccatgattac	*I-isce* I sequence (bold), Homology arm H2 (italic)
Amp t-*I-Sce*I-F	aaa**attaccctgttatcccta**cacgttaagggattttggtcat	*I-isce* I sequence (bold)
OriT-R6K-*I-Sce*I-R	aaa**attaccctgttatcccta**	*I-isce* I sequence (bold)
Identification HA^c^		
BAC-F	gagaacagaaaagaaagcgcgt	
BAC-R	cgcagccacagaaaagaaacga	

^a^Primers used for the construction of the BAC insertion vector. The restriction are marked in italics. ^b^Primers used for modification of donor plasmid pRThGA. ^c^Primers used for verification HA gene insertion into pBAC-C-KCE base on MAGIC.

### Construction of a C-KCE BAC clone

**Figure 2:**
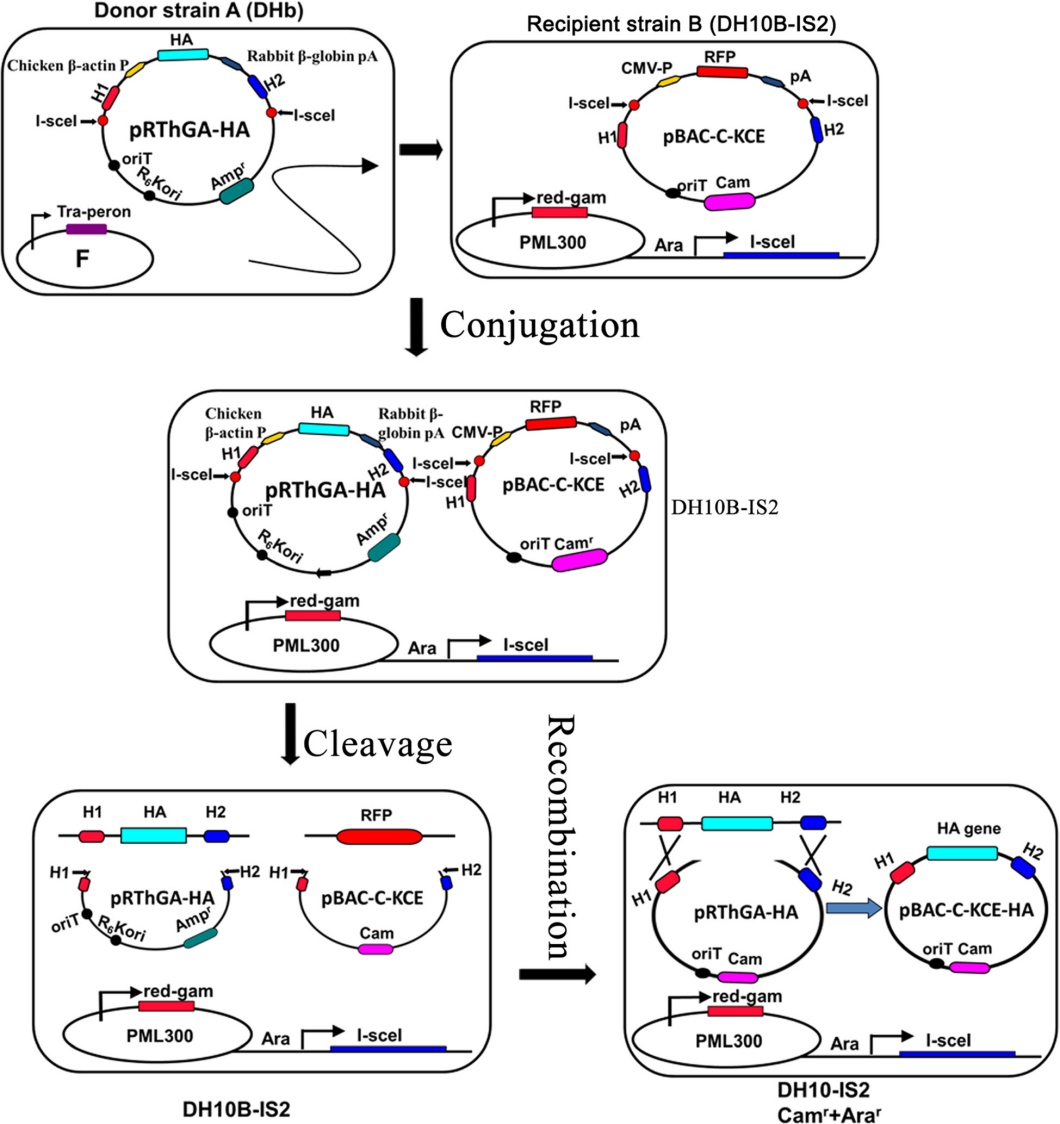
**Procedures for generating pBAC-C-KCE-HA base on MAGIC strategy.** The donor and recipient plasmids were generated as described in the text, then transformed into the donor strain DH10b and recipient strain DH10B-IS2. The DNA fragments of HA and its expression cassette in the donor vector (pRTHGA-HA), and RFP and its expression cassette in the recipient vector (pBAC-C-KCE) were both cut down by an intron-encoding rare endonuclease *I-Sce*I induced by 0.2% w/v L-arabinose, and then the recombination events were intermediated by the red and gam recombinase induced by 0.2% w/v rhamnose. Then, the recombinant vector pBAC-C-KCE-HA was generated.

After C-KCE was incubated to CEF cells at a multiplicity of infection (MOI) of 50 for 2 h at 37 °C, pBlue-lox-gB-amp linearized with *Pac*I (Figure 2C) was transfected by calcium phosphate precipitation. When the complete cytopathic effect was observed, the total supernatant was harvested. The infected virus was diluted and then plated on the fresh CEF, and overlaid with DMEM-FBS containing 0.5% methylcellulose. When red fluorescent plaques were observed, plaque-purification was carried out as previously described [25] to obtain a fluorescent plaque population, termed vBAC-C-KCE. Circular viral DNA was extracted from CEF by the method of Hirt [27]. Approximately 5 μg of genomic DNA was used to electroporate *DH10B-IS2* with 0.1 cm cuvettes under the following conditions: 1.5 kV, resistance of 200 Ω, and capacitance of 25 μF. The plasmid pBAC-C-KCE was isolated from chloramphenicol-resistant colonies using QIAprep miniprep kit (Qiagen), and transfected into CEF by the calcium phosphate precipitation method.

### Generating the recombinant pBAC-C-KCE-HA vector by MAGIC and deleting the BAC vector

*E. coli* DH10b containing the donor vector pRThGA1-HA was grown in LB broth containing 100 μg/mL ampicillin. The recipient strain DH10B-IS2, containing the plasmid pML300 and recipient plasmid pBAC-C-KCE, was grown in LB broth containing 50 μg/mL spectinomycin, 34 μg/mL chloramphenicol, 100 μg/mL streptomycin, and 0.2% w/v glucose overnight. The recipient strain was washed twice with two units of LB the next day. The donor and recipient strains were separately diluted to 1:25, 1:50, 1:100, or 1:200 with LB containing 0.2% w/v rhamnose, and grown at 30 °C for 2 h to an A600 of 0.15–0.25. The donor and recipient strains were mixed to a ratio of 1:1 based on their A600 in the presence of 0.2% w/v L-arabinose. The mixture was incubated at 37 °C for 2 h without shaking, and then for a further 2 h with shaking. The recombinant culture was diluted at a ratio of 1:100, plated on selective plates containing 34 μg/mL chloramphenicol, 100 μg/mL streptomycin, and 0.2% w/v L-arabinose, and finally incubated at 42 °C overnight. The positive clone was named pBAC-C-KCE-HA. To excise the BAC vector sequence, pC-KCE-BAC–HA was co-transfected with pCAGGS-NLS/cre into CEF. The excised BAC vector named C-KCE-HA virus was purified by plaque.

### Confirmation of the expression of the H5 HA gene in CEF infected with the C-KCE-HA

HA protein expression in the recombinant C-KCE-HA was evaluated by immunofluorescence (IFA) and Western blot. For IFA, the CEF grown on coverslips in six-well plates were infected at an MOI of 1 with C-KCE or C-KCE-HA. The monoclonal antibody (mAb) against HA (previously prepared in our laboratory) or polyclonal antibody (pAb) against UL23 (previously prepared in our laboratory) were used as primary antibodies. The secondary antibodies were fluorescein isothiocyanate-conjugated goat anti-rabbit (for HA detection) or anti-mouse (for UL23 detection) IgG (Santa Cruz Biotechnology, CA, USA). The CEF nuclei were stained with 4’-6-diamidino-2-phenylindole (DAPI). The cells were observed using laser scanning confocal microscopy (Carl Zeiss, Zena, Germany). The results were analyzed using software Image J (NIH, USA). For western blot analysis, HA expression was analyzed in CEF in six-well plates infected with C-KCE-HA and C-KCE at an MOI of 1. mAb against HA, pAb against UL23, and mAb against GAPDH (Santa Cruz Biotechnology, CA, USA) for the control were used as primary antibodies. Goat HRP-conjugated anti-rabbit or anti-mouse IgG were used as secondary antibodies. The bands were visualized using Electro-Chemi-Luminescence kit (Thermo, USA) according to the manufacturer’s instructions.

### Stability and growth properties of the recovered virus C-KCE-HA

To analyze the genetic stability of the foreign gene in the recombinant virus, the virus was sequentially grown on primary CEF for 30 passages, and viral DNA was extracted and analyzed after each passage using HA-specific PCR (Table 1). To compare the growth of C-KCE and C-KCE-HA, a multi-step growth kinetic assay was performed and the plaque sizes were measured as previously described [28]. In order to observe the size of the plaques, the cells were stained with crystal violet, and the plaques were readily visible where the cells were destroyed by viral infection.

### Animal experiments

SPF ducks were obtained from the Harbin Veterinary Research Institute, China. Total of 405 one-month-old SPF ducks were used for our studies. Six animal experiments were conducted to evaluate the safety, immunogenicity, and protective efficacy of the C-KCE-HA vaccine against challenge with both homologous and heterologous HPAIV H5N1 and DEV.

For safety experiments, three groups of ducks (five per group) were subcutaneously inoculated with 10^7^ PFU of C-KCE-HA (a recommended dose for DEV vaccine is 10^5^ PFU), C-KCE, or PBS as a control.

For immunogenicity experiments against DEV, we subcutaneously inoculated three groups of ducks (five per group) with 10^5^ PFU of C-KCE-HA, C-KCE, or PBS as a control. At 0, 1, 2, 3, and 4 weeks post-vaccination (pv), serum samples were obtained weekly from all ducks to screen the neutralizing (NT) antibody against DEV.

For clinical protection of C-KCE-HA against virulent DEV challenge**,** three groups of ducks (twenty per group) were inoculated subcutaneously with 10^5^ PFU of C-KCE or C-KCE-HA, whereas naive control ducks were inoculated with PBS. Then, each groups of ducks were randomly subdivided into four groups (five per group). The ducks were challenged with a 100-fold 50% duck lethal dose (DLD_50_) of HB/10 by intramuscular injection either at 3 days, 1 week, 2 weeks, or 12 weeks pv.

To test the serological responses against HPAIV XN/07 and HM/06 stimulated by immunized C-KCE-HA in ducks, ducks randomly divided into three groups of ducks (five per group) received one immunization subcutaneously with 10^5^ PFU of C-KCE-HA, C-KCE, or PBS as a negative control. Serum samples were obtained weekly for 12 weeks from all the groups to monitor the hemagglutination inhibition (HI) and neutralization antibodies.

To detect the cellular response primed by immunized C-KCE-HA in ducks, three groups of ducks (twenty per group) were subcutaneously inoculated with C-KCE-HA (10^5^ PFU), C-KCE (10^5^ PFU), or PBS (control). At 1, 4, 12, and 36 weeks pv, five ducks of each group were sacrificed humanely. Their spleens were collected to evaluate the cellular immune responses.

To evaluate the clinical protection of C-KCE-HA against HPAIV XN/07, 120 ducks were randomly divided into 12 groups (ten per group). Four groups of ducks were inoculated subcutaneously with 10^5^ PFU of C-KCE-HA, and eight groups were inoculated with 10^5^ PFU of C-KCE (four groups) or PBS (four groups) as a negative control. Each treatment ducks were then intramuscularly challenged with a 100-fold DLD_50_ of XN/07 at 3 days, 1 week, 2 weeks, or 12 weeks pv. Three ducks in each group were humanely euthanized on day 3 post-challenge (pc), and their organs, including lung, spleen, kidney, and brain, were collected to determine virus titration in 10-day-old SPF embryonated chicken eggs as previously described [29]. Oropharyngeal and cloacal swabs were collected on days 3, 5, and 7 pc for virus titration in eggs. Ducks were monitored daily for signs of disease and death pc for 2 weeks. The animal experimental design about evaluating the clinical protection of C-KCE-HA against HPAIV XM/06 is the same as the XN/07 mentioned above.

### Serologic tests and virus titration

NT antibody against HB/10 was tested in DEF as previously described [12]. Serum samples were obtained to monitor HA-specific antibodies via HI assays using chicken red blood cells. The NT antibody against homologous XN/07 and heterologous HM/06 of AIV was determined in MDCK cells as described previously [30]. Each swab was washed in 1 mL of PBS with 200 μg/mL penicillin and 200 μg/mL streptomycin. One gram of each organ was collected and mixed into 1.0 mL of PBS, homogenized, and clarified by centrifugation. Virus titration was conducted in 10-day-old SPF embryonated chicken eggs, and calculated using the method of Reed and Muench [31].

### Interferon-γ (IFN-γ) ELISpot assay

Duck spleens were homogenized and washed with Hank Balanced Salt Solution media. Gey solution was added to remove the red blood cells. Splenocytes in Complete Tumor Medium were added into a 96-well (1 × 10^5^–2 × 10^5^ cells per well) plate pretreated with 70% ethanol and coated with anti-duck IFN-γ mAb. Cells were re-stimulated with the HA 518 epitope conserved in both H5N1 viruses used in this study, including currently circulating avian and human H5N1 viruses, and some H9N2 viruses [32]. The cultures were incubated at 37 °C and 5% CO_2_ for 48 h, and developed according to an ELISpot protocol (TSZ, USA). Spots were counted using an AID ViruSpot Reader (Cell Technology, Inc.).

### Statistical analysis

All experiments were reproducible and performed in triplicate. Statistical analyses were conducted by a one-way ANOVA test to compare the data of the difference groups using GraphPad Prism version 5.0 (GraphPad Software, La Jolla, CA, USA). *p*-values of < 0.05 were considered statistically significant.

### Laboratory facility

All experiments related to HPAIV were conducted in a bio-security level-3 facility.

## Results

### Establishing a full-length C-KCE clone harboring mini-F plasmid sequences

**Figure 3:**
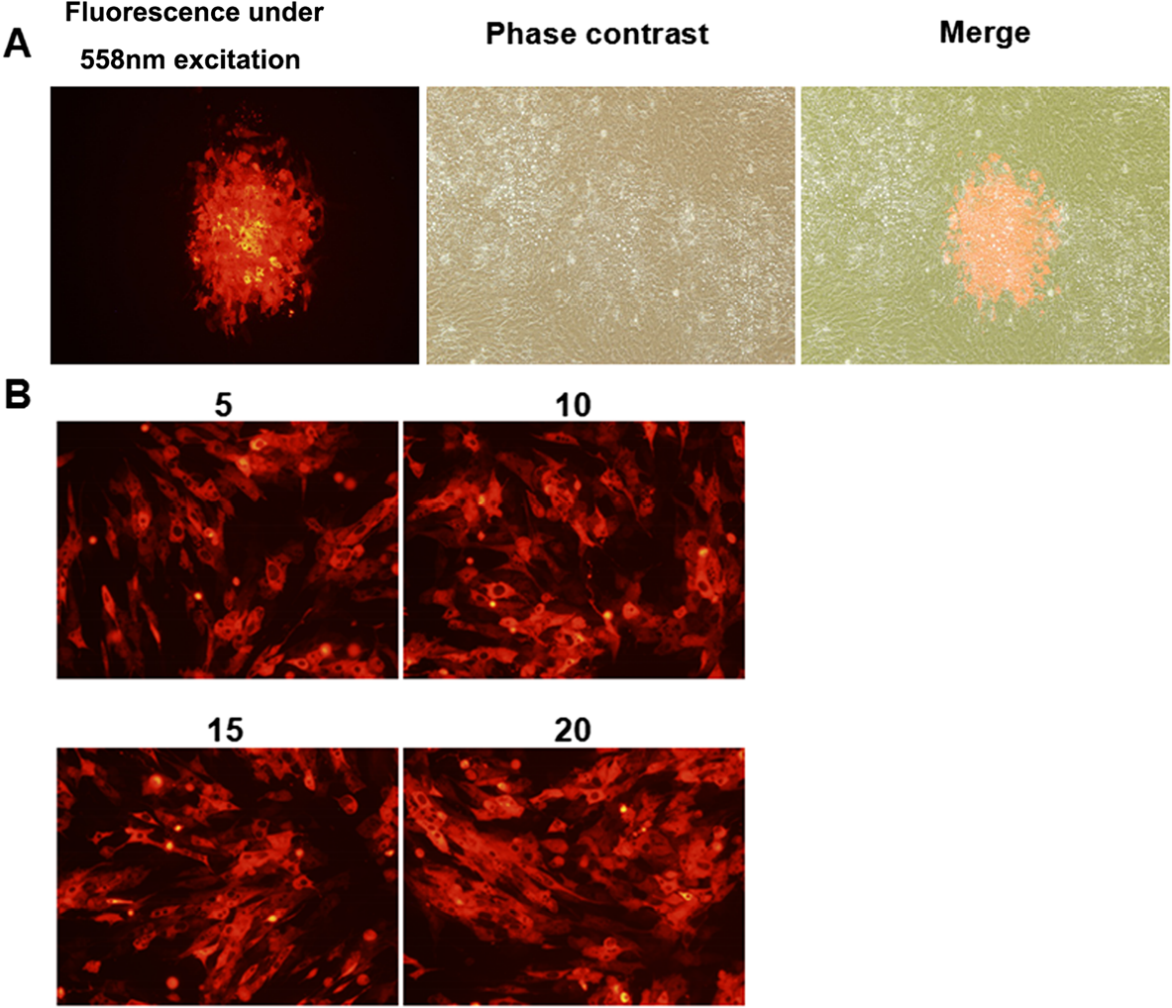
**Characterization of the recombinant virus BAC-C-KCE.** **(A)** Plaques of recombinant virus vBAC-C-KCE and parental virus C-KCE were shown under green light excitation, phase contrast, or merge (400×). **(B)** Investigation of the genetic stability of vBAC-C-KCE. The numbers represent the passages of the vBAC-C-KCE (400×). Red fluorescent protein as a selection marker was observed under fluorescence microscopy.

**Figure 4:**
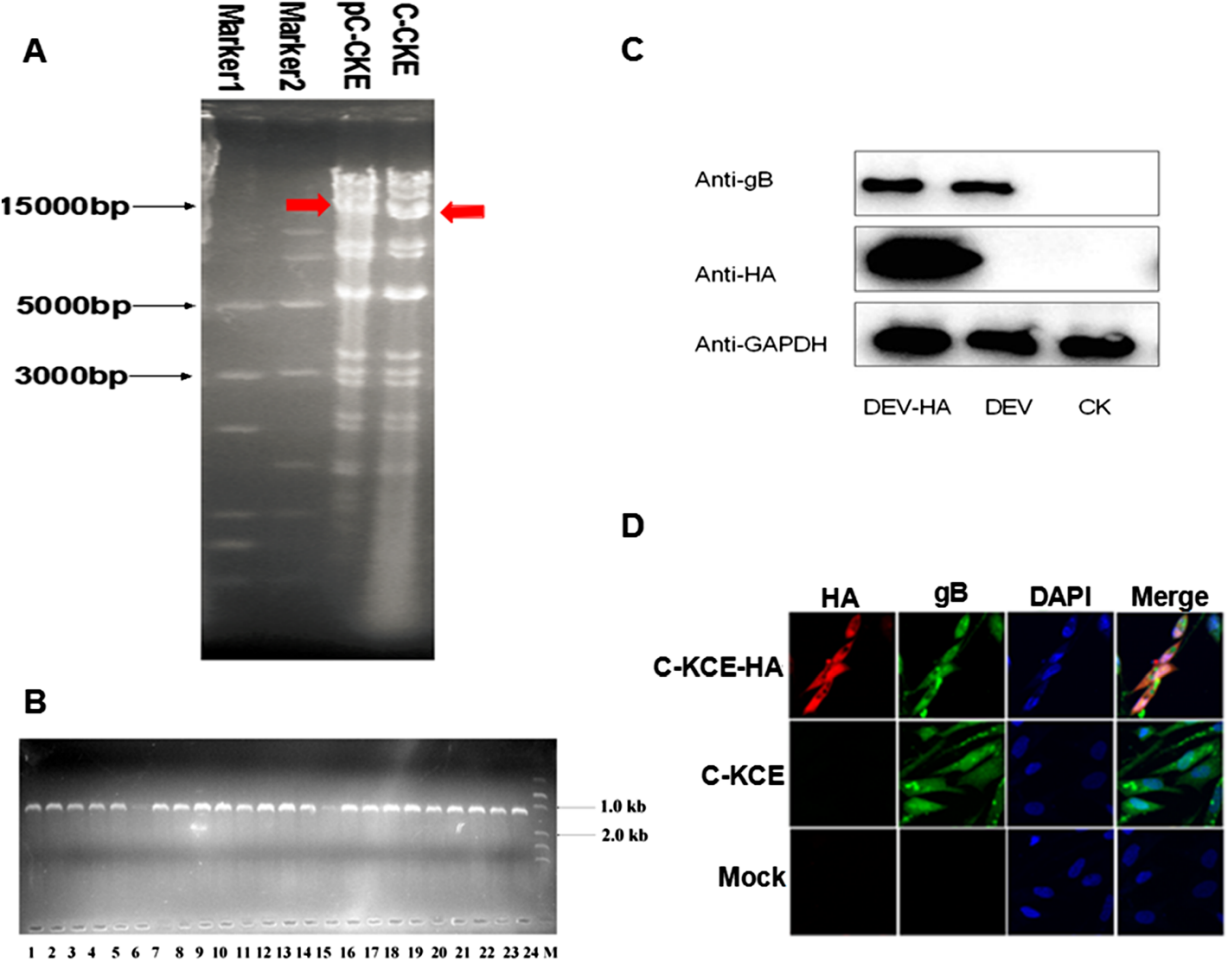
**Efficiency of HA gene insertion in pBAC-C-KCE base on MAGIC and characterization of the recombinant virus C-KCE-HA.** **(A)** The genome BAC-C-KCE and C-KCE were isolated and digested with *BamH* I and separated with a 0.8% agarose gel. The red arrowhead shows that the band in line 3 are bigger than the band in line 4 (that is 8.3 kb by analyzing the genome using DNAman tool). The sizes of a molecular weight marker (15 000-bp and 5000-bp marker, Transgen) are given. **(B)** Detection of the HA gene insertion in pBAC-C-KCE by PCR. The marker used was DL15000. **(C)** Detection the expression of HA protein in C-KCE-HA-infected CEF by Western blotting. **(D)** Confirmation of the expression of HA protein in C-KCE-HA-infected CEF using immunofluorescence.

Establishing a full-length C-KCE clone in *E. coli* first requires the insertion of a BAC vector into the viral genome (Figure 1A). Thus, the BAC vector was inserted into a large junction of the gB and UL26 genes in the C-KCE genome (Figure 1B). The resulting virus, vBAC-C-KCE, was plaque-purified based on the expression of RFP (Figure 3A). The virus was passaged in CEF for 20 rounds to evaluate the genetic stability of the purified vBAC-C-KCE (Figure 3B). The circular viral DNA from the vBAC-C-KCE-infected CEF was extracted, and transformed into the *E. coli* strain DH10B-IS2. pBAC-C-KCE (Figure 1D) was isolated using a QIAprep miniprep kit and transfected into CEF. At 5 days after transfection, approximately 90% of the clones resulted in cytopathic effects. Two clones were selected randomly and sequenced in Shanghai Southgene Technology Co., Ltd. We found that one of the two clones covered the entire 158 kb C-KCE genome (GenBank ID: KF263690.1). Restriction fragment length polymorphisms (RFLP) of C-KCE and BAC-C-KCE were analyzed to confirm that a full-length C-KCE BAC clone was indeed generated (Figure 4A). This clone was termed pBAC-C-KCE for subsequent studies. The findings confirm that BAC was stably inserted into the gB and UL26 junction region in a site-specific manner.

### Rapid generation of the C-KCE-HA vaccine encoding H5N1 HA

The red fluorescent gene, CMV promoter, and poly A were entirely replaced by the HA gene and its cassette. Thus, the HA gene was expressed under the control of the chicken β-actin promoter. The colonies were randomly selected and screened by PCR. A 1.2 kb fragment was amplified from the positive recombinant plasmid pBAC-C-KCE-HA (Figure 1E), and the region around the recombination site was further confirmed by sequencing. Among the randomly selected 24 colonies, 22 contained the expected recombinant vector pBAC-C-KCE-HA (Figure 4B). The efficiency of the positive clone was as high as 90%. BAC vector sequences were flanked by two direct orientation *Loxp* sequences. pBAC-C-KCE-HA was co-transfected with pCAGGS-NLS/cre to remove the BAC vector sequence. After Cre-mediated removal of the BAC vector sequences, non-fluorescent plaques appeared and were collected. The resulting C-KCE-HA virus (Figure 1F) without the BAC backbone was plaque-purified and then confirmed by PCR (data not shown). Based on our experimental results, we found that the HA gene could be inserted into pBAC-C-KCE via MAGIC. Moreover, the C-KCE-HA virus, whose BAC backbone was eliminated, was successfully generated by *Cre/Lox*p–mediate recombination, and only the 34 bp *Loxp* sequences were positioned in the C-KCE-HA genome.

### Biological characterization and stability of the rescued C-KCE-HA recombinant viruses

To assess the genetic stability and growth kinetics of C-KCE-HA, the virus was grown on CEF sequentially for 30 passages. The viral DNA was extracted and analyzed after each passage using HA-specific PCR (data not shown).

To compare the growth of C-KCE and C-KCE-HA, the assays of multi-step growth kinetics and measurements of plaque size were performed. The growth kinetics of C-KCE-HA was similar to that of C-KCE (Additional file 1A). The plaque sizes were also similar (Additional file 1B). These results reveal that the HA gene was stably inserted into the C-KCE genome, and exerted no adverse reaction on C-KCE replication in vitro. The expression of the HA protein was determined by western blot and IFA. As expected, the cells infected with C-KCE-HA reacted well. Strong signals were visualized using ECL detection reagents with mAb for HA or pAb for UL23. By contrast, the parental virus C-KCE reacted well with mAb for UL23, and the blank cells did not react with either of the antibodies (Figure 4C). The results of IFA matched those of western blot well. As shown in Figure 4D, diffused HA and UL23 expression were observed, indicating that the HA protein was expressed in the C-KCE-HA-infected CEF. These results also imply that the E protein was robustly expressed during C-KCE-HA replication.

### Virulence and immunogenicity evaluation of C-KCE-HA

In our safety experiments, all the ducks remained healthy during the observation period, demonstrating that the insertion of the HA gene did not increase the virulence of the vector C-KCE virus.

To evaluate whether inserting a foreign gene influences the immunogenicity of the parental virus C-KCE, serum samples were obtained weekly for four weeks from all ducks vaccinated with C-KCE-HA, C-KCE, or PBS to screen the NT antibody, a marker of immunogenicity, against HB/10. The NT antibody titers of the PBS-inoculated groups were lower than 3 log_2_ and considered negative (Additional file 2). By contrast, the NT antibody titers of three ducks exceeded 2^3^ in C-KCE and C-KCE-HA vaccinated groups at one week pv. The NT antibody titers of four ducks reached 2^4^ at two weeks pv, but the titers of the two groups started to drop rapidly since then; however, they were still higher than those of the control group (Additional file 2).

Although the NT antibody titers primed by C-KCE or C-KCE-HA were low and short-lived, no significant difference was observed, indicating that the insertion of the HA gene did not change the immunogenicity of the parental virus C-KCE.

### Clinical protection of C-KCE-HA against virulent DEV challenge

**Figure 5:**
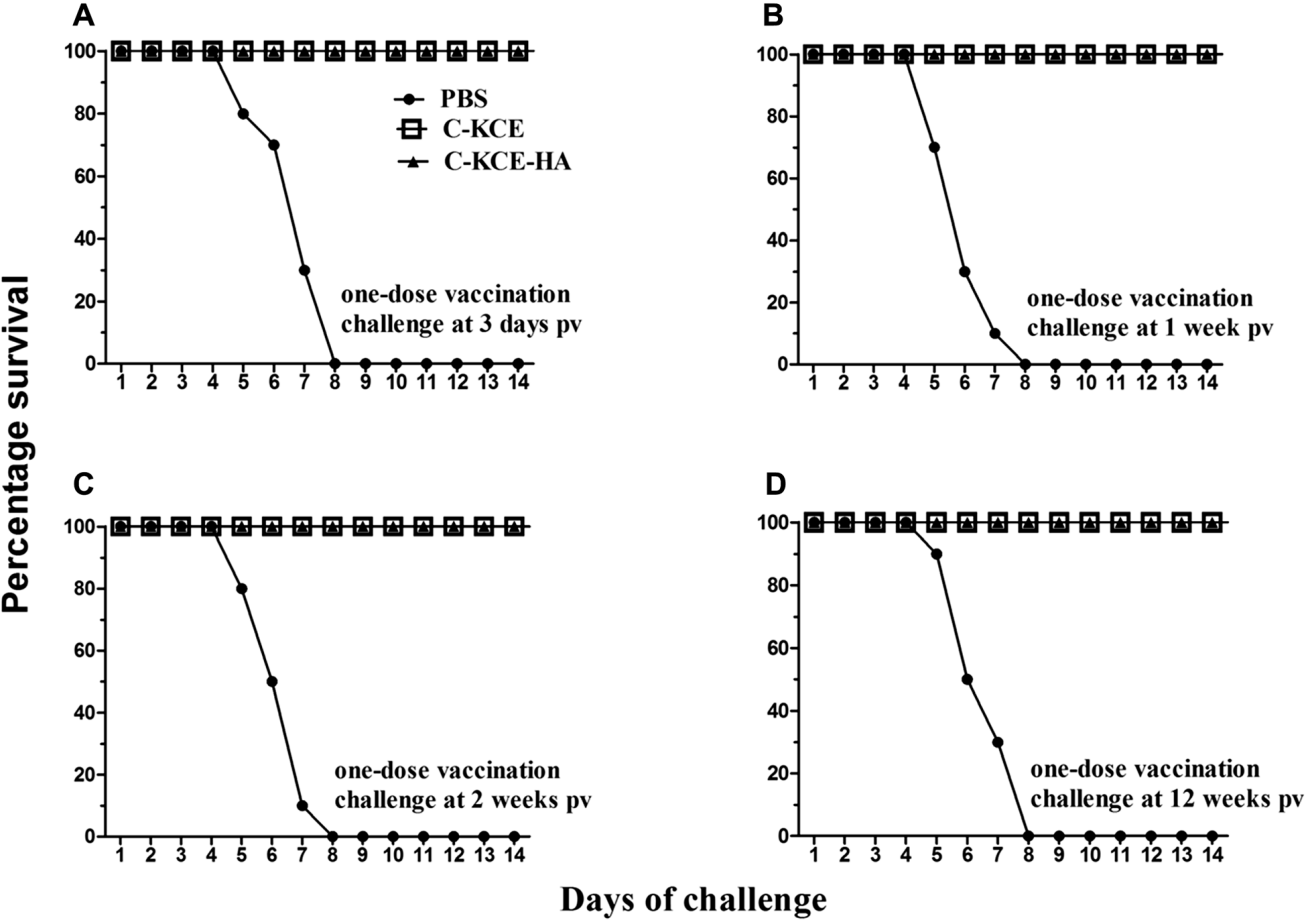
**Conferred protection from immunization of duck with C-KCE-HA vaccine against challenge with virulent DEV challenge.** Ducks were inoculated subcutaneously with 10^5^ PFU of C-KCE-HA, 10^5^ PFU of C-KCE or with PBS as a control, then intramuscularly challenged with 100-fold DLD_50_ HB/10 at 3 days **(A)**, 1 week **(B)**, 2 weeks **(C)**, or 12 weeks **(D)** post-vaccination (pv), respectively. Ducks were monitored daily for 2 weeks after challenge.

Animal experiments were conducted to examine the effect of the inserted exogenous gene on the protective efficacy of the parental virus C-KCE, and evaluate the efficacy of the C-KCE-HA vaccine against HB/10 challenge. The ducks immunized with C-KCE or C-KCE-HA survived the lethal challenge regardless of when they were challenged with HB/10 in our experiments. However, two ducks that received C-KCE-HA and were challenged on day 3 pv (Figure 5) showed slight and transient symptoms, including polydipsia and slight loss of appetite, at the beginning of the experimental period. Conversely, the PBS-inoculated ducks showed severe symptoms and succumbed to infection within 8 days (Figure 5). The protective efficacy of C-KCE-HA and C-KCE against lethal DEV challenge showed no difference. These results demonstrate that the insertion of the HA gene did not alter the protective efficacy of C-KCE.

### Induction of antibody response in C-KCE-HA-vaccinated ducks

**Figure 6:**
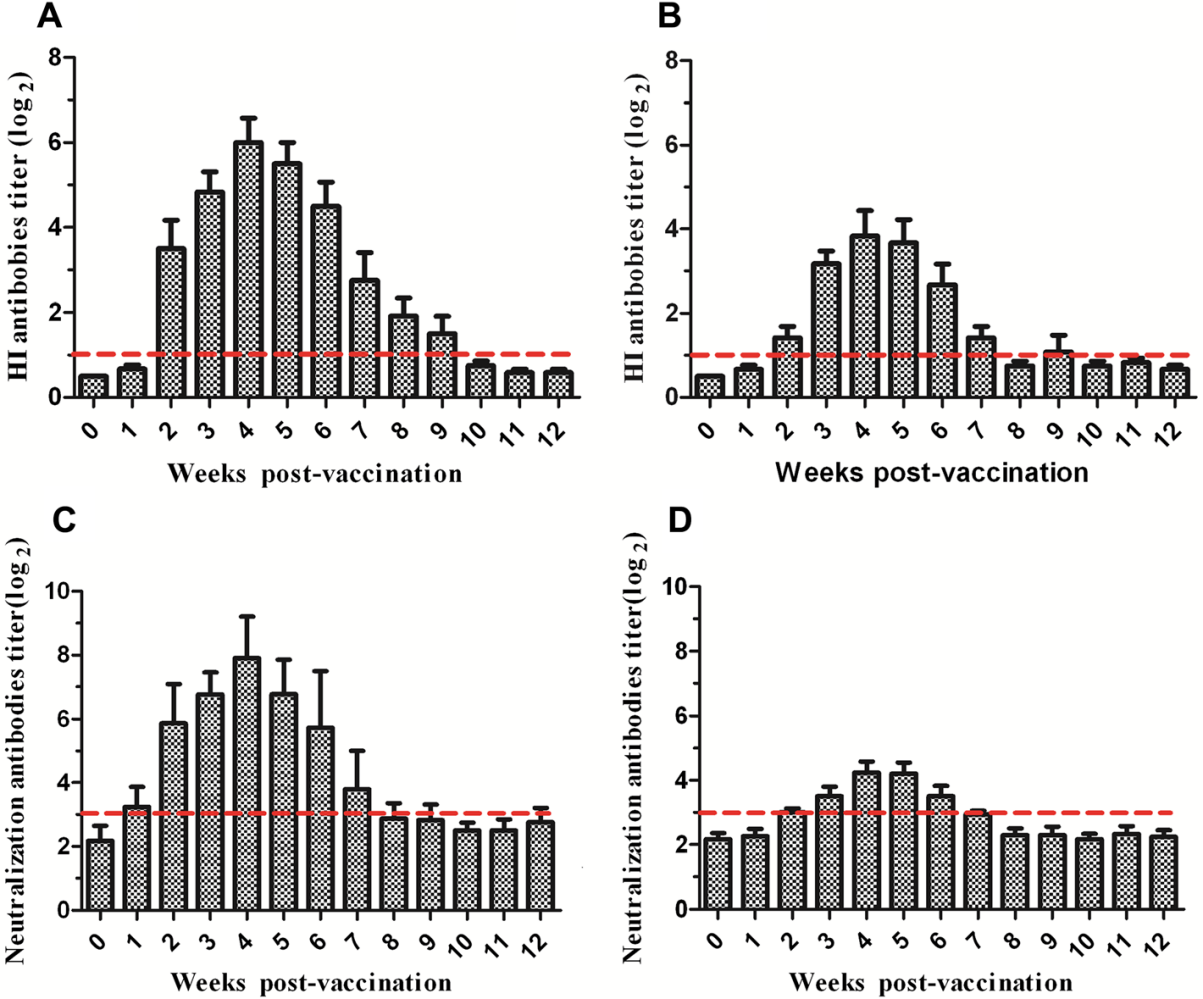
**Serological response against homologous and heterologous avian influenza virus strains in duck immunized with C-KCE-HA.** Ducks were inoculated subcutaneously with 10^5^ PFU of C-KCE-HA. Sera were collected from ducks weekly for HI antibody and NT antibody detection. **(A)**. HI responses were assessed against the homologous XN/07 virus. **(B)**. HI responses were assessed against the heterologous HM/06 virus. **(C)**. NT responses were assessed against the homologous XN/07 virus. **(D)**. NT responses were assessed against the heterologous HM/06 virus. Dotted lines indicate the thresholds for a positive response.

Antigenic drift is associated more with HA compared with other genes [10]. The HA gene of XN/07 shares approximately 95% identity with HM/06 (Additional file 3). The HA gene of XN/07 shares approximately 95% identity with HM/06 (Additional file 3). To determine whether the serological responses induced by C-KCE-HA vaccine can cross-react with more recent H5N1 viruses isolated from ducks, XN/07 and HM/06 were analyzed by HI and NT. No detectable HI and NT were observed against XN/07 antigens tested in ducks that received mock vaccination (data not shown). Tests were carried out on the sera of C-KCE-HA-immunized ducks to detect the HI antibody against XN/07. The earliest detection of an immune response to XN/07 was at week 2. The antibody level started to increase at week 3 with titers of 2^4^ ± 2^2^, and it peaked at week 4 with titers of 2^6^ ± 2^1^. However, the antibody level began to decline at week 5. Ultimately, the HI antibody was not detected from any duck at 12 weeks pv until the end of our analysis (Figure 6A). The HI antibody responses against HM/06 were consistent with those observed in XN/07, but the HI level was approximately 2^2^ titers lower (Figure 6B).

Sera from vaccinated ducks were also evaluated using the NT assay to detect functional antibodies with neutralization activity against the XN/07 (Figure 6C) and HM/06 (Figure 6D) viruses. The trends of NT and HI antibody responses in the C-KCE-HA-inoculated groups were similar, but the levels of the NT antibody were higher than those of the HI antibody at all time points tested (Figure 6). These results demonstrate that vaccination with C-KCE-HA induced cross-reactive antibody responses, and primarily induced antibody responses specific to the delivery of the HA strain.

### Cellular response to C-KCE-HA virus vaccination

**Figure 7:**
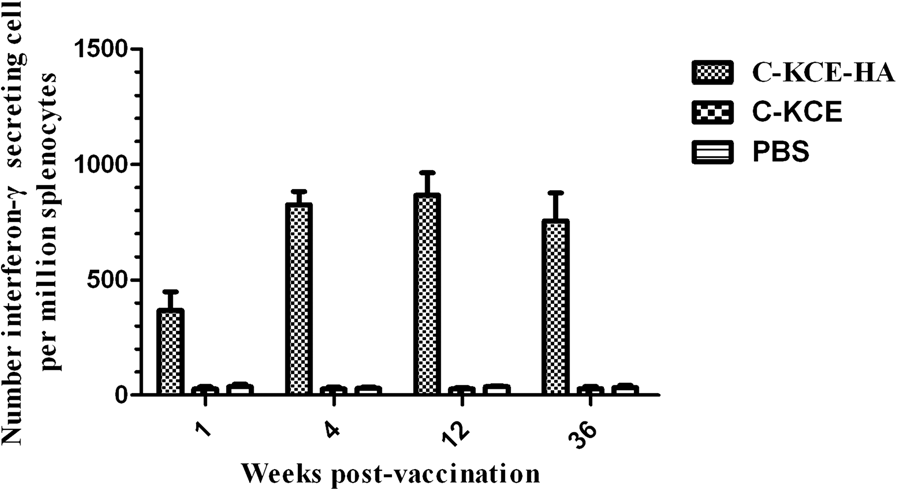
**Induction of HA-518-epitope-specific and IFN-γ-secreting spleen cells in ducks immunized with C-KCE-HA vaccine.** HA-specific responses of splenocytes taken 1, 4, 12, 36 weeks post-vaccination as determined by IFN-γ-ELISPOT assay inducing HA-518-epitope-specific. Data represent means standard errors of the mean of triplicate determinations for a minimum of three ducks per group.

IFN-γ ELISpot assays were performed to evaluate whether C-KCE-HA can prime cellular immune responses. As expected, ducks that received the C-KCE-HA vaccine demonstrated significantly increased numbers of IFN-γ-secreting cells in spleen cells, regardless of the time the spleen cells were stimulated with the conserved HA 518 epitope (Figure 7). By contrast, numbers of IFN-γ-secreting cells in the groups of C-KCE and PBS were limited. These data demonstrate that C-KCE-HA vaccination robustly generated cellular immune responses to HA.

### Vaccine efficacy against lethal H5N1 AIV challenge in ducks

To evaluate whether C-KCE-HA can induce cross-protection against H5 AIV, we challenged the vaccinated ducks with the homologous AIV XN/07 and heterologous AIV HM/06. When we challenged the ducks that received a dose of C-KCE-HA with a 100-fold DLD_50_ of AIV, they were fully protected from challenge with both homologous (Table 2) and heterologous (Table 3) AIV H5N1. The challenged virus was neither recovered from any organs tested nor detected in the oropharynx and cloacae (Tables 2 and 3). All the ducks remained healthy during the observation period. In the control groups of ducks challenged with XN/07 and HM/06 viruses, both viruses were detected in the lung, kidney, spleen, and brain, with titers ranging from 4.3 to 8.6 log_10_ 50% egg infectious doses (lgEID_50/_g) (Additional file 4). All the ducks shed the virus through both the oropharynx and cloacae on day 3 and died after days 4 to 5 pc (Tables 2 and 3). These data indicate that a single dose of 10^5^ PFU of C-KCE-HA could induce solid cross-protection against homologous and heterologous H5N1 AIV challenge. Thus, it completely blocked replication and shedding of the challenged virus at early stages in ducks.

**Table 2:** **Protective efficacy of C-KCE-HA against H5N1 lethal virus XN/07 challenge in ducks**

**Challenge virus**	**Challenge time pv**	**Vaccine**	**Virus isolation from the swabs (shedding/total log**_**10**_ **EID**_**50**_ **/mL)**						**No. surviving/total**
			**Day 3 pc**		**Day 5 pc**		**Day 7 pc**		
			**Oropharyngeal**	**Cloacal**	**Oropharyngeal**	**Cloacal**	**Oropharyngeal**	**Cloacal**	
XN/07	1 week	C-KCE-HA	0/7	0/7	0/7	0/7	0/7	0/7	7/7
		C-KCE	7/7(3.1 ± 0.6)	5/7(2.4 ± 0.5)	—	—	—	—	0/7
		PBS	7/7(4.2 ± 0.5)	6/7(3.8 ± 0.4)	—	—	—	—	0/7
	3 weeks	C-KCE-HA	0/7	0/7	0/7	0/7	0/7	0/7	7/7
		C-KCE	7/7(2.7 ± 0.6)	6/7(2.4 ± 0.6)	—	—	—	—	0/7
		PBS	7/7(4.3 ± 0.7)	7/7(4.3 ± 0.7)	—	—	—	—	0/7
	12 weeks	C-KCE-HA	0/7	0/7	0/7	0/7	0/7	0/7	7/7
		C-KCE	7/7(2.7 ± 0.7)	7/7(2.6 ± 1.1)	—	—	—	—	0/7
		PBS	7/7(2.1 ± 0.9)	4/7(1.8 ± 1.2)	—	—	—	—	0/7
	36 weeks	C-KCE-HA	0/7	0/7	0/7	0/7	0/7	0/7	7/7
		C-KCE	7/7(3.1 ± 0.3)	6/7(2.1 ± 0.5)	—	—	—	—	0/7
		PBS	7/7(2.7 ± 0.7)	7/7(1.8 ± 0.6)	—	—	—	—	0/7

One-month-old SPF ducks were used in these studies. Groups of seven ducks were inoculated subcutaneously with 10^5^ PFU of C-KCE-HA, 10^5^ PFU of C-KCE or with PBS as a control. They then were challenged intramuscularly with 100-fold DLD_50_ homologous (XN/07) AIVs at 1 week, 3 weeks, 12 weeks or 24 weeks post-vaccination (pv). Oropharyngeal and cloacal swabs were collected on days 3, 5, and 7 post-challenge (pc) and titrated in SPF eggs. All of the ducks in C-KCE and PBS groups died within 5 days. The horizontal line indicates that the animals had died by that time point.

**Table 3:** **Protective efficacy of C-KCE-HA against H5N1 lethal virus HM/06 challenge in ducks**

**Challenge virus**	**Challenge time pv**	**Vaccine**	**Virus isolation from the swabs (shedding/total log**_**10**_ **EID**_**50**_ **/mL)**						**No. surviving/total**
			**Day 3 pc**		**Day 5 pc**		**Day 7 pc**		
			**Oropharyngeal**	**Cloacal**	**Oropharyngeal**	**Cloacal**	**Oropharyngeal**	**Cloacal**	
HM/06	1 week	C-KCE-HA	0/7	0/7	0/7	0/7	0/7	0/7	7/7
		C-KCE	7/7(2.1 ± 0.9)	6/7(2.5 ± 0.3)	—	—	—	—	0/7
		PBS	7/7(3.4 ± 1.2)	7/7(3.8 ± 0.4)	—	—	—	—	0/7
	3 weeks	C-KCE-HA	0/7	0/7	0/7	0/7	0/7	0/7	7/7
		C-KCE	7/7(4.1)	7/7(3.3 ± 0.5)	—	—	—	—	0/7
		PBS	7/7(3.6 ± 0.7)	7/7(2.5 ± 1.1)	—	—	—	—	0/7
	12 weeks	C-KCE-HA	0/7	0/7	0/7	0/7	0/7	0/7	7/7
		C-KCE	7/7(2.6 ± 0.8)	7/7(3.1 ± 0.6)	—	—	—	—	0/7
		PBS	7/7(2.9 ± 0.6)	6/7(2.3 ± 1.1)	—	—	—	—	0/7
	36 weeks	C-KCE-HA	0/7	0/7	0/7	0/7	0/7	0/7	7/7
		C-KCE	7/7(2.5 ± 0.6)	5/7(2.8 ± 0.3)	—	—	—	—	0/7
		PBS	7/7(2.7 ± 0.9)	6/7(2.4 ± 0.6)	—	—	—	—	0/7

One-month-old SPF ducks were used in these studies. Groups of seven ducks were inoculated subcutaneously with 10^5^ PFU of C-KCE-HA, 10^5^ PFU of C-KCE or with PBS as a control. They then were challenged intramuscularly with 100-fold DLD_50_ heterologous (HM/06) AIVs at 1 week, 3 weeks, 12 weeks or 24 weeks post-vaccination (pv). Oropharyngeal and cloacal swabs were collected on days 3, 5, and 7 post-challenge (pc) and titrated in SPF eggs. All of the ducks in C-KCE and PBS groups died within 5 days. The horizontal line indicates that the animals had died by that time point.

## Discussion

Several infectious pathogens, particularly HPAIV H5N1, can seriously threaten the progression of the duck industry. Vaccines are the most effective method to control these pathogens, and several approaches are being taken worldwide to develop vaccines against these pathogens. However, all of them can pose both practical and immunological challenges [33]. Therefore, a novel vaccine must meet a number of criteria, including low production cost, ease of production, high production yield, and ease of administration. An appealing strategy for improving the immunology of the virus vaccine in a pratical manner involves the use of a live *Anatid herpesvirus* which can deliver foreign antigens of other viruses and can therefore serve as a dual vaccine. Thus, we established a BAC clone of DEV attenuated strain C-KCE. The HA gene of HPAIV H5N1 was inserted into the C-KCE genome through MAGIC. Although C-KCE-HA contained an HA gene and its cassette, insertion exerted no adverse effect on C-KCE replication in vitro, and it did not alter the pathogenicity and immunogenicity of C-KCE in vivo. After a single immunization, C-KCE-HA induced humoral immune and cellular immune responses to HA. As early as one week pv, ducks provided solid protection against challenge with homologous and heterologous H5N1 virus without viral shedding, clinical signs, and death. Therefore, the use of C-KCE-HA could simultaneously prevent two deadly infectious diseases, namely, AI and DEV, with a single live virus vaccine.

Viral vectors have been widely explored for developing vaccines. Fowlpox virus, varicella-zoster virus, pseudorabies virus, turkey herpesvirus, adenovirus, baculovirus, and Newcastle disease virus-based vectors [11,32,34-38] are the most extensively studied viral vectors. Some recombinant vaccines have even been granted licenses by some governments, and they are currently utilized for preventing the spread of pathogens. Attenuated DEV vaccine strains, including C-KCE and clone03, are ideal avian vaccine vector candidates because these viruses can induce long-lasting protection against DEV in ducks, and they have a natural host range limited to avian species [39].

Herpesvirus mutagenesis has come to rely on BAC and recombinant technology to generate recombinant viruses. Construction of the BAC of C-KCE can be used to develop a bivalent vaccine as well as facilitate the study of DEV pathogenesis. For example, the virulent strain 2085 is the first established infectious BAC used to evaluate the function of the gC gene [25]. Two general approaches to infectious clone design could be applied to establish a BAC clone of the C-KCE. In one approach, the BAC vector could be inserted into a viral gene dispensable for viral growth and pathogenesis. In another approach, the BAC vector was inserted into the gene junction of the C-KCE genome. The expression of the neighboring genes could not be interfered in this junction. Currently, the full genomes of the three strains of DEV are available in GenBank [15,40,41]. Molecular characterization of the genome of DEV is similar to other herpesvirus type 1. Those areas have been proven to be suitable for foreign gene insertion, including the sites within gG, gB, US2, and gD [42-45]. The insertion site of the foreign gene could alter the immunogenicity and vaccine efficacy of recombinant DEV [39]. Hence, we inserted a mini-F vector into the gB and UL26 gene junction, which is the longest among all the junctions within the C-KCE genome (454 bp). The stability of vBAC-C-KCE was confirmed by assaying vBAC-C-KCE, which was serial passaged 20 times, in CEF. This finding indicates that the junction of C-KCE was an ideal location for foreign gene insertion.

We accurately and efficiently inserted the HA gene into pBAC-C-KCE via MAGIC. Notably, the efficiency of the positive clone was approximately 90%. MAGIC has been explored in many viruses, such as adenoviruses and baculovirus [21,46]. In contrast to other traditional systems, this novel strategy is highly efficient, time-efficient, and uses a low level of background nonrecombinants to construct a recombinant virus. The vaccine strains for AI control have been frequently updated; AIV have undergone rapid genetic evolution, and expanded their host range and virulent properties in mammals [12]. Thus, BAC-C-KCE based on MAGIC could be considered for the development of the AI vaccine against newly emerging AIV in ducks. Once the HA sequence of an emerging AIV is known, a bivalent vaccine can be rapidly generated. For example, the recent emergence of the AIV (H7N9) strain in poultry and its subsequent transmission to humans in China have raised great concerns about the potential pandemic spread of lethal diseases [47]. Utilizing MAGIC, we successfully constructed and obtained the recombinant vaccine expression of HA of H7N9 within two weeks of acquiring the HA gene (data not shown).

Generally, the expression level has two important determinants, including the inserted gene itself and its promoter. Previous studies have demonstrated that the expression of HA in the pCAGGS vector can display higher levels than that in other vectors [11,15]. We reasoned that the amount of HA expression was critical to the efficacy of C-KCE-HA for inducing protective neutralization antibodies against AIV. To this end, we also selected the chicken β-actin promoter driving a high-level of protein expression across a wide range of species and cell types. Indeed, our results show that HA protein was robustly expressed during C-KCE-E replication, and the protection efficiency of C-KCE-HA also improved [39].

The neutralization antibody against influenza virus is the hallmark of protective immunity [32]. Serological data revealed that the antibodies could be barely detected at 1 and 12 weeks pv. Of note, our challenge studies showed that the C-KCE-HA provided protection from a lethal challenge with homologous virus XN/07 and early isolate HM/06, which is antigenically different. Thus, in addition to neutralizing, antibodies play a role in this protection but T-cell responses, which have been shown to aid in virus clearance, also contribute to this protection [32]. Clearly, the HA protein is the major surface glycoprotein of the influenza virus; it not only induces HA-specific antibodies, but also stimulates cellular responses [36]. Our study builds upon these findings, going one step further in trying to understand the role of the T-cell response to the C-KCE-HA influenza virus HA vaccine. The presence of heterotypic H5N1 protection without a sufficient humoral neutralizing response, further reinforced by the ability of the C-KCE-HA vaccine to fully protect the immunized duck, strongly suggests a complementary role for the cellular response to its humoral counterpart. Moreover, the provision of long-term immunity with a single dose of C-KCE-HA had obvious benefits to layers and breeder flocks [39]. Overall, the C-KCE-based vaccine could be utilized to offset traditional inactivated AIV vaccines.

Our findings highlight the potential of a BAC-C-KCE-vector-based delivery system, which offers stockpiling options for the development of a pandemic influenza vaccine. In addition to insertion of HA alone, due to the huge capacity of the BAC system, viral NP and M2 can be inserted to generate a universal influenza vaccine [48] against newly emerging antigenic drift and shift variants. Similarly, immunogenic genes of other pathogens, such as duck hepatitis virus, duck tembusu virus, and AIV H9N2, could also be inserted at the same time to generate a polyvalent vaccine. We expect that the application of this novel BAC-C-KCE platform to develop a series of vaccines in the near future will greatly decrease those pathogens in poultry.

## Additional files

Additional file 1:
**Comparsion of the growth between C-KCE and C-KCE-HA.** (A). Multi-step growth kinetics of C-KCE-HA and C-KCE in CEF cells. Data were shown for the indicated time points after infection with an MOI of 0.01. Titers are given as plaque forming units in 0.1 mL. Means of virus titers as determined by three independent experiments are shown; standard deviations are shown with the error bars. (B). Plaque phenotype of C-KCE-HA and C-KCE in CEF cells. After titration of viruses, cells were fixed in 4% paraformaldehyde and stained with crystal violet. Additional file 2:
**Humoral immune response against virulent DEV in ducks vaccinated with C-KCE-HA or its parental strain C-KCE.** Groups of 5 ducks were inoculated subcutaneously with 10^5^ PFU of C-KCE-HA, C-KCE or with PBS as a control. Sera were collected range from 0 to 4 weeks to detect the NT antibody against virulent DEV in DEF cells. NT antibody titers for ducks are expressed as a log_2_. Dotted lines indicate the thresholds for a positive response. Additional file 3:
**Phylogenetic relationships of the HA genes of H5N1 AIV.** The tree includes AIV that were isolated from some provinces in China during 2004 to 2010. The phylogenetic tree was generated with the MEGA (version 5.0) by using the neighbor-joining algorithm and based on bootstrap values of 1000. The HA gene donor virus for the recombinant vaccine generation and the challenge viruses used in this study are marked in red. Additional file 4:
**Replication of challenge virus in ducks.** Homologous and heterologous H5N1 replication in the organs of ducks that were vaccinated with C-KCE-HA. Groups of three ducks were inoculated subcutaneously with 10^5^ PFU of C-KCE-HA, C-KCE or with PBS as a control. They then were challenged with homologous (XN/07) or heterologous (HM/06) AIV intramuscularly at 1 week, 3 weeks, 12 weeks or 36 weeks pv. Day 3 after challenge, the ducks were euthanized and their organs were harvested for virus titration in eggs. Data represent means ± standard deviations of log_10_ EID_50_s. The backslash indicates that the challenge virus was not detected by that time point.
