# Supplementary material for: Efficient strategy for constructing duck enteritis virus-based live attenuated vaccine against homologous and heterologous H5N1 avian influenza virus and duck enteritis virus infection
Source: Vet Res. 2015 Apr 16;46(1):42. doi: 10.1186/s13567-015-0174-3 (PMC4397706; doi:10.1186/s13567-015-0174-3)

A Multi-step growth kinetics of C-KCE-HA and C-KCE in CEFs

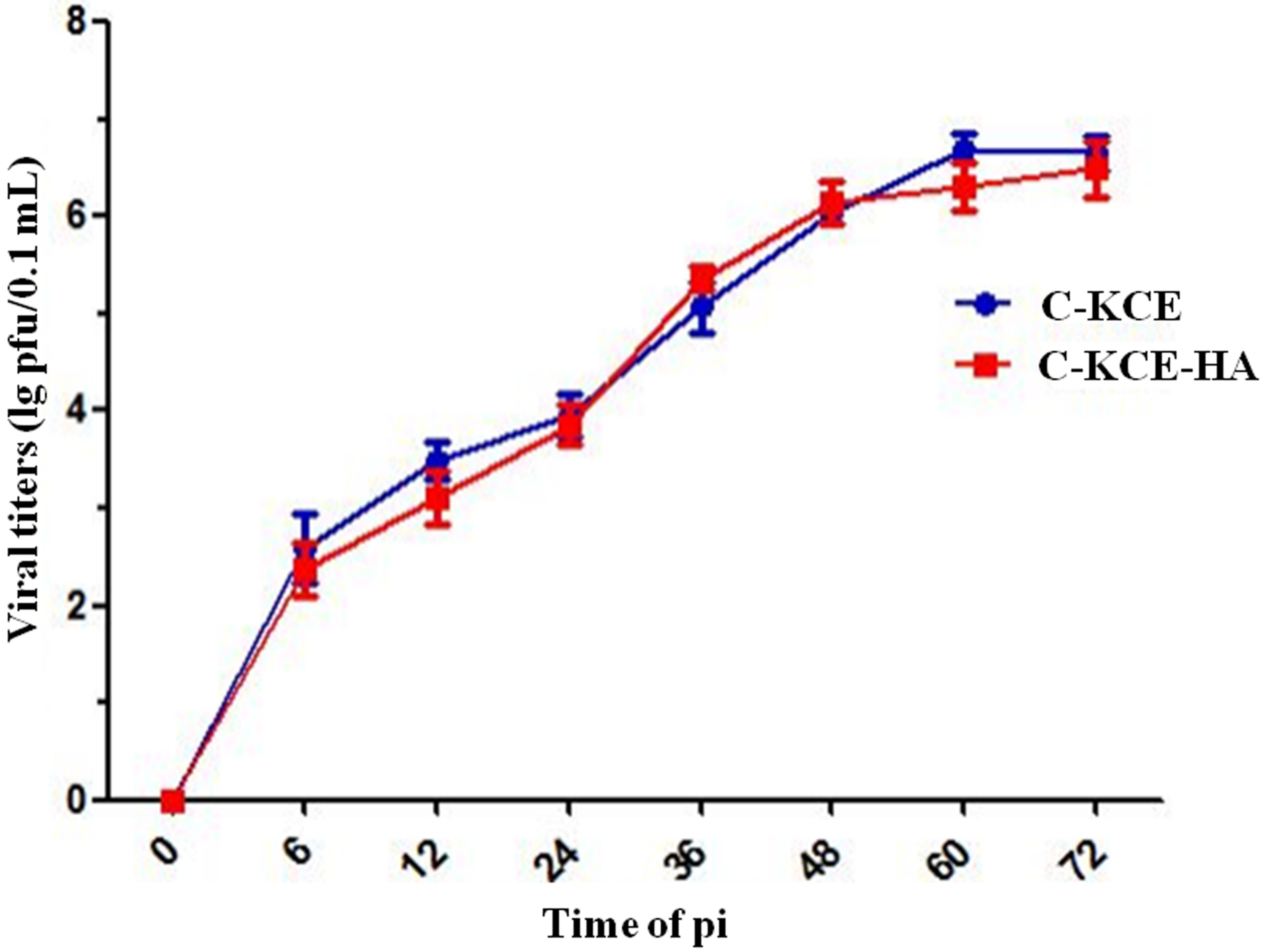

B Plaque phenotype of C-KCE-HA and C-KCE in CEFs

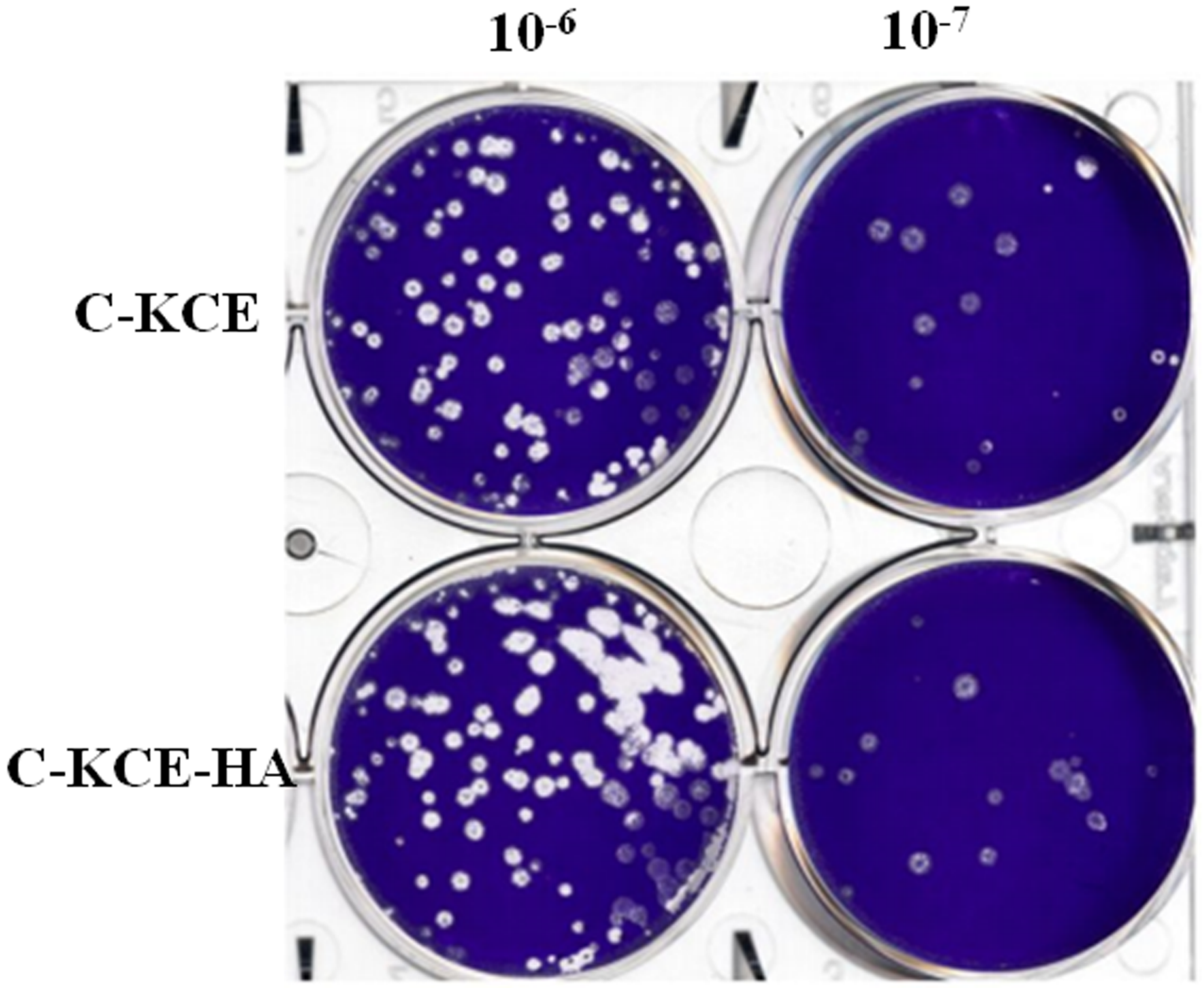

Supplement: Additional file 1: — Comparsion of the growth between C-KCE and C-KCE-HA. (A). Multi-step growth kinetics of C-KCE-HA and C-KCE in CEF cells. Data were shown for the indicated time points after infection with an MOI of 0.01. Titers are given as plaque forming units in 0.1 mL. Means of virus titers as determined by three independent experiments are shown; standard deviations are shown with the error bars. (B). Plaque phenotype of C-KCE-HA and C-KCE in CEF cells. After titration of viruses, cells were fixed in 4% paraformaldehyde and stained with crystal violet. [file 13567_2015_174_MOESM1_ESM.pdf]
