# Supplementary figures and images for: Efficient strategy for constructing duck enteritis virus-based live attenuated vaccine against homologous and heterologous H5N1 avian influenza virus and duck enteritis virus infection
Source: Vet Res. 2015 Apr 16;46(1):42. doi: 10.1186/s13567-015-0174-3 (PMC4397706; doi:10.1186/s13567-015-0174-3)

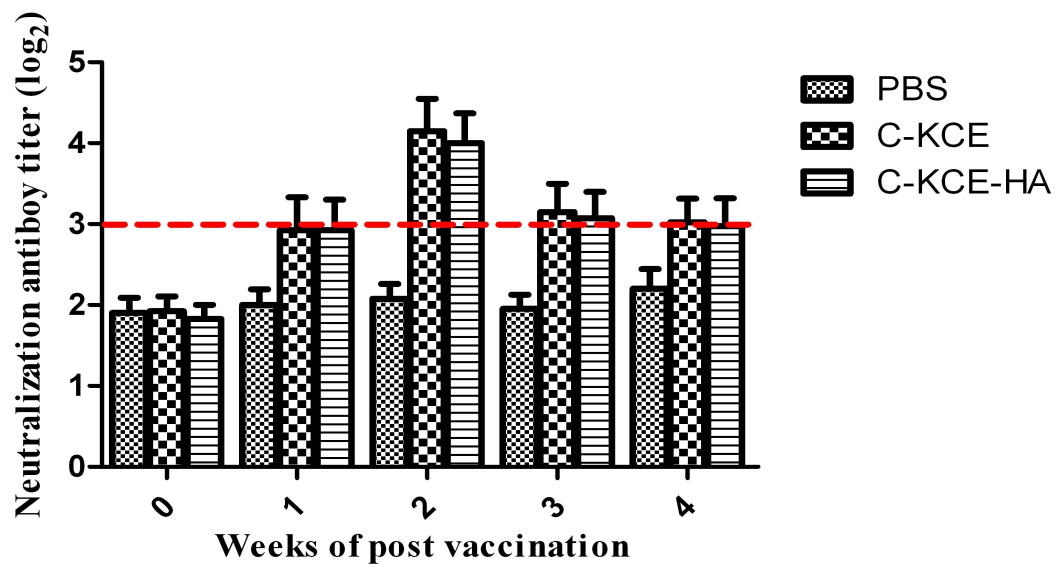

Supplement: Additional file 2: — Humoral immune response against virulent DEV in ducks vaccinated with C-KCE-HA or its parental strain C-KCE. Groups of 5 ducks were inoculated subcutaneously with 105 PFU of C-KCE-HA, C-KCE or with PBS as a control. Sera were collected range from 0 to 4 weeks to detect the NT antibody against virulent DEV in DEF cells. NT antibody titers for ducks are expressed as a log2. Dotted lines indicate the thresholds for a positive response. [file 13567_2015_174_MOESM2_ESM.pdf]

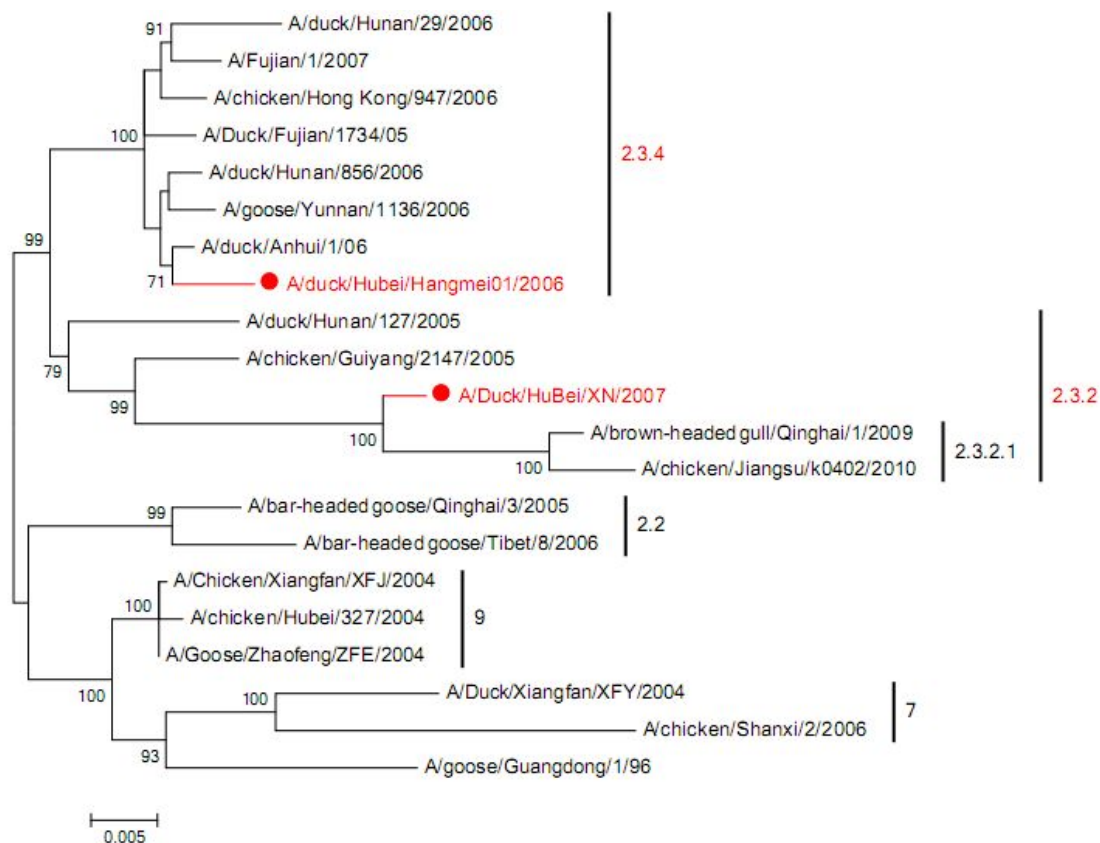

Supplement: Additional file 3: — Phylogenetic relationships of the HA genes of H5N1 AIV. The tree includes AIV that were isolated from some provinces in China during 2004 to 2010. The phylogenetic tree was generated with the MEGA (version 5.0) by using the neighbor-joining algorithm and based on bootstrap values of 1000. The HA gene donor virus for the recombinant vaccine generation and the challenge viruses used in this study are marked in red. [file 13567_2015_174_MOESM3_ESM.pdf]
