# Supplementary material for: Efficient strategy for constructing duck enteritis virus-based live attenuated vaccine against homologous and heterologous H5N1 avian influenza virus and duck enteritis virus infection
Source: Vet Res. 2015 Apr 16;46(1):42. doi: 10.1186/s13567-015-0174-3 (PMC4397706; doi:10.1186/s13567-015-0174-3)
Supplement: Additional file 4: — Replication of challenge virus in ducks. Homologous and heterologous H5N1 replication in the organs of ducks that were vaccinated with C-KCE-HA. Groups of three ducks were inoculated subcutaneously with 105 PFU of C-KCE-HA, C-KCE or with PBS as a control. They then were challenged with homologous (XN/07) or heterologous (HM/06) AIV intramuscularly at 1 week, 3 weeks, 12 weeks or 36 weeks pv. Day 3 after challenge, the ducks were euthanized and their organs were harvested for virus titration in eggs. Data represent means ± standard deviations of log10 EID50s. The backslash indicates that the challenge virus was not detected by that time point. [file 13567_2015_174_MOESM4_ESM.docx]

| Challenge  virus | Challenge  time pv | vaccine | Virus replication in the organs in the ducks on 3 days pv ( lg10EID_50_/g) | | | |
| --- | --- | --- | --- | --- | --- | --- |
|  |  |  | Brain | Lung | Spleen | Kidney |
| XN/07 | 1 week | C-KCE-HA | / | / | / | / |
|  |  | C-KCE | 6.7 ± 0.5 | 7.1 | 6.3 ± 0.5 | 7.0 ± 0.6 |
|  |  | PBS | 7.6 | 7.2 ± 1.4 | 6.6 ± 1.2 | 6.5 ± 1.5 |
|  | 3 weeks | C-KCE-HA | / | / | / | / |
|  |  | C-KCE | 7.4 ± 0.5 | 7.1 | 6.4 ± 1.1 | 7.0 ± 0.7 |
|  |  | PBS | 7.6 ± 0.7 | 7.0 ± 1.5 | 6.0 ± 1.4 | 6.8 ± 1.4 |
|  | 12 weeks | C-KCE-HA | / | / | / | / |
|  |  | C-KCE | 7.8 ± 0.8 | 7.3 ± 1.1 | 7.0 ± 0.8 | 6.5 ± 1.0 |
|  |  | PBS | 7.4 ± 0.6 | 6.8 ± 1.4 | 7.6 | 7.0 ± 1.1 |
|  | 36 weeks | C-KCE-HA | / | / | / | / |
|  |  | C-KCE | 7.4 | 7.6 ± 0.8 | 7.2 ± 0.3 | 6.8 ± 1.2 |
|  |  | PBS | 7.8 ± 0.8 | 7.3 | 7.0 ± 0.8 | 6.5 ± 1.0 |
| HM/06 | 1 week | C-KCE-HA | / | / | / | / |
|  |  | C-KCE | 6.5 | 6.4 ± 0.3 | 7.6 ± 0.5 | 5.5 ± 1.1 |
|  |  | PBS | 7.8 ± 0.9 | 7.2 ± 1.4 | 6.6 ± 1.2 | 6.6 ± 1.5 |
|  | 3 weeks | C-KCE-HA | / | / | / | / |
|  |  | C-KCE | 6.4 ± 0.5 | 7.2 ± 0.8 | 6.8 | 6.4 ± 0.7 |
|  |  | PBS | 7.7 ± 0.9 | 7.5 ± 0.8 | 6.5 ± 1.1 | 6.6 ± 1.4 |
|  | 12 weeks | C-KCE-HA | / | / | / | / |
|  |  | C-KCE | 6.6 ± 0.5 | 6.3 | 6.2 ± 0.8 | 6.0 ± 1.1 |
|  |  | PBS | 7.6 ± 0.8 | 7.3 ± 1.2 | 6.7 ± 1.1 | 6.6 ± 0.8 |
|  | 36 weeks | C-KCE-HA | / | / | / | / |
|  |  | C-KCE | 7.3 ± 0.3 | 6.8 ± 0.9 | 7.2 | 6.7 ± 0.6 |
|  |  | PBS | 7.8 ± 0.6 | 7.1 ± 0.9 | 7.3 ± 0.6 | 6.8 |
